# A Modified Obturation Strategy Using the Piston Technique With a Calcium Silicate–Based Sealer in the Management of an Immature Necrotic Mandibular Second Molar: A Case Report

**DOI:** 10.1155/crid/7042394

**Published:** 2026-08-07

**Authors:** Bruno Calkovsky, Rastislav Juricek, Sarah Kalmanova, Maria Janickova

**Affiliations:** ^1^ Department of Stomatology and Maxillofacial Surgery, Jessenius Faculty of Medicine in Martin, Comenius University Bratislava, Martin, Slovakia

**Keywords:** calcium silicate sealer, open apex, piston technique

## Abstract

Endodontic management of immature permanent teeth with pulp necrosis is challenging due to open apices and thin root walls. This case report describes an alternative obturation strategy—the piston technique—in which a calcium silicate–based sealer serves as the primary apical sealing material, while thermoplasticized gutta‐percha acts mainly as a pressure‐transmitting medium to facilitate its apical displacement. A 14‐year‐old patient presented with tenderness to percussion of an immature mandibular second molar with an open apex and periapical radiolucency. A diagnosis of pulp necrosis with symptomatic apical periodontitis was established. Treatment was performed in two visits. After chemo‐mechanical debridement, calcium hydroxide was placed as an intracanal medicament. At the second visit, obturation was achieved using the piston technique, in which calcium silicate–based sealer is combined with thermoplasticized gutta‐percha, allowing apical sealing without the need for an artificial apical barrier. The tooth was subsequently restored. At follow‐up, the patient was asymptomatic, and radiographic evaluation showed evidence of an adequate apical seal and periapical healing. The combination of a calcium silicate–based sealer and thermoplasticized gutta‐percha, as used in the piston technique, may represent a clinically feasible obturation strategy in selected cases for immature necrotic posterior teeth when regenerative procedures are not indicated.

## 1. Introduction

Endodontic management of immature permanent teeth with pulp necrosis is clinically demanding due to open apices [1], thin dentinal walls, and the risk of inadequate apical sealing [2]. Conventional approaches such as apexification with calcium hydroxide or MTA apical barrier techniques [3] are effective but may be technically challenging, particularly in posterior teeth with limited access [4]. Calcium silicate–based sealers have been introduced as bioactive materials that may facilitate obturation when regenerative procedures are not indicated [5, 6].

In this report, the piston technique refers to a modified obturation approach in which gutta‐percha is used primarily as a pressure‐transmitting medium rather than as the main core filling material. In this concept, thermoplasticized gutta‐percha is used primarily to generate operator‐controlled apically directed pressure, actively displacing a calcium silicate–based sealer toward the apical region. Unlike conventional warm vertical condensation [7], in which gutta‐percha constitutes the main bulk of the filling, this technique relies on the hydraulic movement of the sealer to achieve adequate apical sealing.

In practical terms, the manufacturer‐supplied syringe‐mounted applicator tip is positioned loosely 3–6 mm short of the apical foramen. The calcium silicate–based sealer is then delivered into approximately the apical two‐thirds of the canal. Thermoplasticized gutta‐percha is subsequently introduced into the coronal third using a cordless backfill device. Careful vertical compaction of the gutta‐percha is then performed with minimal pressure, with the intention of transmitting force to the sealer and directing it toward the open apex while avoiding uncontrolled extrusion or overfilling. Intraoperative radiographs may be repeated as needed to assess obturation length, density, and sealer adaptation, allowing further cautious compaction or modification of the filling when clinically indicated.

## 2. Case Report

### 2.1. Patient Information

This case report was prepared in accordance with the CARE (CAse REport) guidelines [8].

The patient was a 14‐year‐old Caucasian male, a secondary school student, who presented with pain and sensitivity on biting in the left mandibular posterior region. He also reported food impaction and localized gingival inflammation around the affected tooth. The tooth had been previously treated at another dental clinic, and the patient was referred to our clinic with the temporary restoration in place. The patient was systemically healthy, with no relevant medical conditions, no regular medication use, and a non‐contributory family history without known hereditary or genetic diseases. No psychosocial or lifestyle factors relevant to dental treatment were identified.

Extraoral examination revealed no facial asymmetry, swelling, or lymphadenopathy. Intraoral examination showed a mandibular second molar with deep carious involvement and a temporary restoration. The surrounding gingival tissue was inflamed and partially overlapping the occlusal surface, with localized signs of gingivitis. The tooth was tender to percussion and biting, with no pathological mobility. Adjacent teeth and the surrounding oral mucosa were clinically normal.

### 2.2. Diagnostic Assessment

Diagnostic evaluation comprised a comprehensive clinical examination, assessment of reported symptoms, percussion and biting tests, and radiographic imaging [9]. A panoramic radiograph (Figure 1), obtained at another dental clinic at the time of initial treatment, was first reviewed. A periapical radiograph (Figure 2) was subsequently used to further evaluate root development, apical morphology, and periapical tissues.

**Figure 1:**
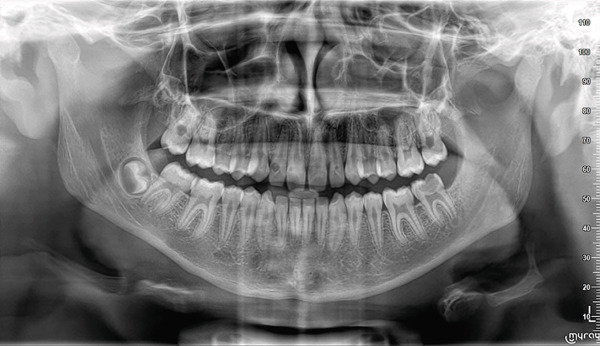
Preoperative panoramic radiograph. Panoramic radiograph obtained at the referring dental clinic at the time of initial treatment. The image shows a mandibular second molar with extremely deep carious involvement and an associated periapical lesion.

**Figure 2:**
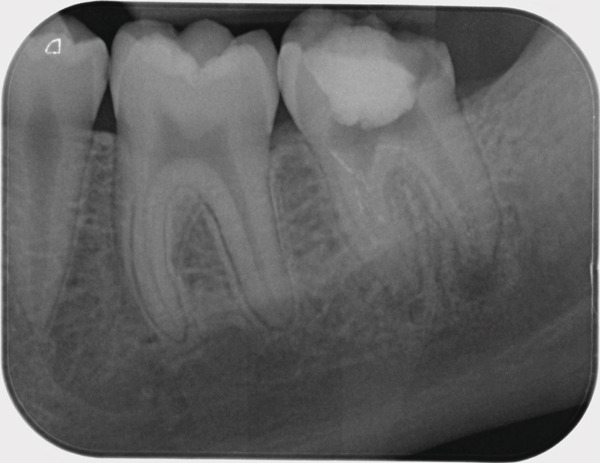
Preoperative periapical radiograph. Periapical radiograph showing the mandibular second molar with carious involvement associated with a temporary coronal restoration and remnants of a temporary root canal filling. A periapical radiolucency is evident, along with significant loss of pericervical dentin and immature root development with open apices.

No financial, language, cultural, or compliance‐related barriers affecting the diagnostic process were identified. The primary challenge was the immature root anatomy with an open apex, which reduced the predictability of conventional obturation techniques.

Based on the clinical findings, radiographic evidence, and the presence of a periapical radiolucency, a diagnosis of pulp necrosis with symptomatic apical periodontitis was established. Informed consent was obtained from the patient’s parents, both verbally and in writing.

The prognosis was considered guarded because of the immature root structure with an open apex and the significant loss of pericervical dentin [10]. Nevertheless, thorough canal disinfection, the use of a bioactive calcium silicate–based sealer [11], the achievement of satisfactory radiographic evidence of apical sealing, and the planned indirect cuspal coverage were expected to positively influence the long‐term outcome [12]. The sequence of diagnostic, therapeutic, restorative, and follow‐up procedures is summarized in Table 1.

**Table 1:** Timeline of case management according to the CARE guidelines.

Time point	Clinical event
Initial presentation	The patient presented with pain and sensitivity on biting in the left mandibular posterior region. Clinical and radiographic examination revealed an immature mandibular second molar with an open apex, periapical radiolucency, and significant loss of pericervical dentin.
Diagnostic assessment	A diagnosis of pulp necrosis with symptomatic apical periodontitis was established based on clinical findings, percussion and biting tests, and radiographic evaluation. Informed consent was obtained from the patient’s parents.
First visit	Chemo‐mechanical root canal debridement was performed under continuous irrigation with 5.25% sodium hypochlorite and activation. No additional apical preparation was performed. Calcium hydroxide was placed as an intracanal medicament.
Second visit, 2 weeks later	Definitive obturation was performed using a calcium silicate–based sealer and thermoplasticized gutta‐percha with the piston technique. The tooth was restored with an interim direct composite resin restoration.
Six‐month follow‐up	The tooth remained asymptomatic and functional. Radiographic evaluation showed signs of periapical healing and partial resorption of the extruded sealer material.
Twelve‐month follow‐up	The patient remained asymptomatic, with normal function and no tenderness to percussion. Radiographic assessment showed further reduction of the periapical lesion, evidence of bone healing, and partial resorption of the extruded sealer material.

### 2.3. Therapeutic Intervention

The therapeutic intervention consisted of non‐surgical endodontic treatment performed using a two‐visit protocol. At the first visit, chemo‐mechanical root canal debridement was carried out [13] under continuous irrigation with 5.25% sodium hypochlorite (Cerkamed Medical Company, Poland), with irrigant activation performed using the EDDY system (VDW GmbH, Munich, Germany) to enhance intracanal irrigant distribution. Given the compromised root structure, no additional apical preparation was performed to avoid further weakening of the root. K‐files up to ISO size 70 were used solely for passive mechanical debridement within the pre‐existing canal space and were passively introduced to the apical extent without intentional enlargement beyond the existing canal dimensions. Calcium hydroxide (Calcipast, Cerkamed Medical Company, Poland) was then placed as an intracanal medicament and left in situ until the second appointment [14].

At the subsequent visit, 2 weeks later, the intracanal medicament was removed and final irrigation was completed using 5.25% sodium hypochlorite and 40% citric acid (Cerkamed Medical Company, Poland), with irrigant activation performed using the EDDY system (VDW GmbH, Munich, Germany) [15].

Definitive root canal obturation was performed using a calcium silicate–based sealer (DiaRoot Sealer, DiaDent Group International, Republic of Korea) in combination with thermoplasticized gutta‐percha (Gutta Percha Bar PLUS, DiaDent Group International, Republic of Korea) applied using the piston technique to achieve apical sealing. The technique involved placement of the sealer into approximately the apical two‐thirds of the root canal, followed by introduction of thermoplasticized gutta‐percha, which was heated and delivered into the coronal third using a cordless backfill device. Vertical condensation of gutta‐percha applied in operator‐controlled manner generated a piston effect, producing apical pressure that directed the sealer toward the apex and resulted in radiographically dense apical sealing. Careful tactile control was maintained to minimize the risk of apical extrusion, and an intraoperative periapical radiograph (Figure 3) was used to verify length and density of the obturation [17].

**Figure 3:**
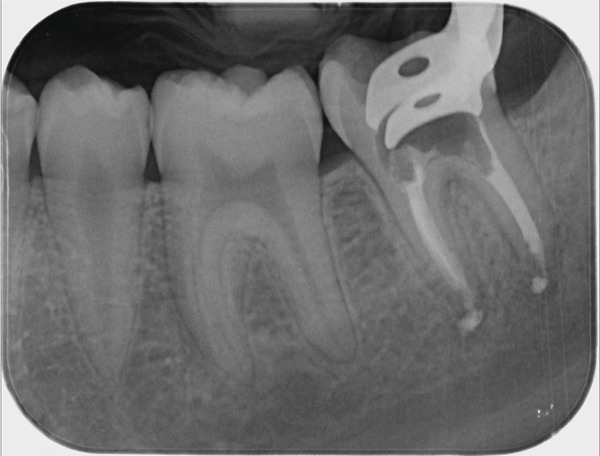
Intraoperative periapical radiograph. Intraoperative periapical radiograph obtained during obturation after vertical condensation pressure was applied to the gutta‐percha to create a piston effect. The radiograph was used to verify the position and homogeneity of the root canal filling materials. Limited sealer extrusion occurred and was considered clinically acceptable [16].

Following endodontic treatment, the tooth was restored with an interim coronal restoration using a direct composite resin in combination with a short fiber–reinforced composite (EverX flow, GC, Japan) and direct coverage of the distal cusps (Figure 4) [18]. The parents were informed that if follow‐up radiographs confirmed favorable healing, indirect cuspal coverage would be recommended to improve the long‐term structural stability of the tooth.

**Figure 4:**
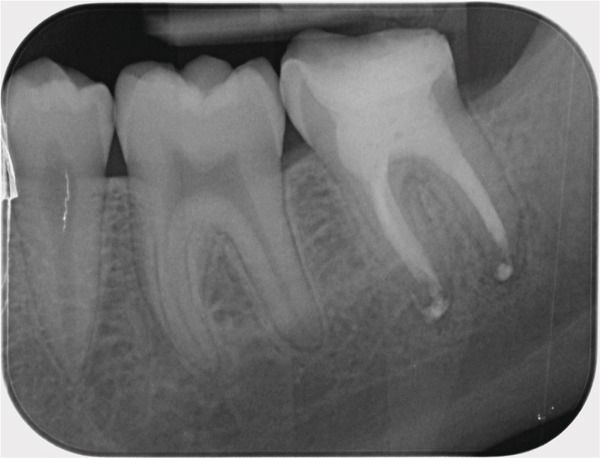
Immediate postoperative periapical radiograph. Immediate postoperative periapical radiograph showing the mandibular second molar after obturation, restored with a composite core build‐up using a short fiber–reinforced composite and direct coverage of the distal cusps.

No systemic pharmacological therapy was required, and no modification to the planned treatment protocol was necessary.

### 2.4. Follow‐Up and Outcomes

At follow‐up visits, the patient reported complete resolution of symptoms, including the absence of pain and discomfort on biting. Clinical examination confirmed normal function, no tenderness to percussion, and healthy surrounding gingival tissues. The treated tooth remained functional and asymptomatic throughout the entire follow‐up period. As part of the definitive restorative plan, indirect cuspal coverage was recommended to ensure long‐term structural stability of the tooth [19].

Radiographic assessment 6 months (Figure 5) and 12 months (Figure 6) showed a progressive reduction of the periapical radiolucency, with evidence of bone healing and maintenance of an adequate apical seal. No radiographic signs of reinfection, root resorption, or other pathological changes were observed.

**Figure 5:**
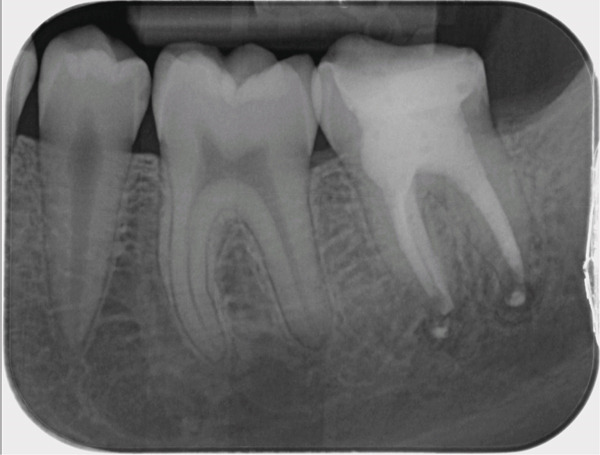
Six‐month follow‐up periapical radiograph. Six‐month follow‐up periapical radiograph showing a persistent periapical lesion of similar extent to that observed immediately after treatment, with radiographic evidence of partial resorption of the extruded sealer material.

**Figure 6:**
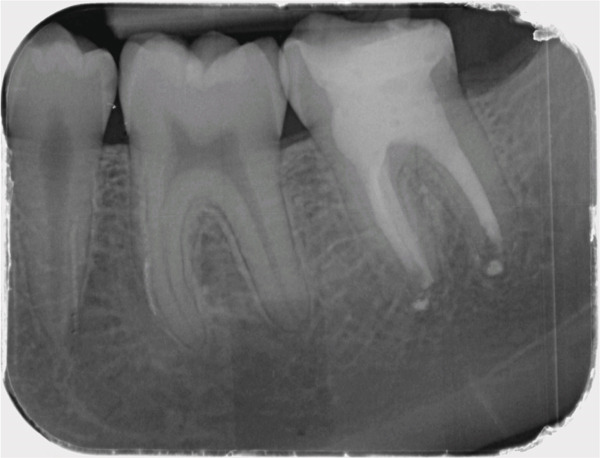
Twelve‐month follow‐up periapical radiograph. Twelve‐month follow‐up periapical radiograph showing a reduction of the periapical lesion and evidence of bone healing and partial resorption of the extruded sealer material.

The patient demonstrated full adherence to the treatment protocol and scheduled follow‐up visits. All procedures were well tolerated, with no discomfort reported beyond the immediate postoperative period. No adverse events, complications, or unanticipated outcomes occurred during treatment or follow‐up.

## 3. Discussion

The main strength of the presented case is the successful management of an immature necrotic mandibular second molar using a conservative, non‐surgical approach that avoided the placement of an artificial apical barrier. In this case, the use of a calcium silicate–based sealer in combination with the piston technique was associated with radiographic evidence of satisfactory sealing in a tooth with an open apex and limited posterior access. A two‐visit protocol with calcium hydroxide dressing supported adequate canal disinfection prior to obturation [20].

The piston technique may be conceptualized as an obturation approach in which thermoplasticized gutta‐percha primarily serves as a pressure‐transmitting medium, facilitating hydraulic displacement of a calcium silicate–based sealer toward the apical region. Unlike conventional warm vertical condensation, in which gutta‐percha is the primary obturation material, this technique utilizes gutta‐percha mainly to drive sealer movement. In contrast to single‐cone techniques relying on passive sealer flow, this approach introduces operator‐controlled pressure to enhance sealer adaptation in the apical area.

Management of immature permanent teeth with pulp necrosis traditionally includes long‐term calcium hydroxide apexification, MTA apical barrier techniques, and regenerative endodontic procedures. Although calcium hydroxide apexification is effective, it is associated with prolonged treatment time and an increased risk of root fracture [21]. MTA apical barrier techniques offer predictable apical closure but can be technically demanding, particularly in posterior teeth with limited access. Regenerative endodontic procedures may represent a biologically based alternative; however, their predictability in molars with complex root canal anatomy remains limited.

In the present case, regenerative endodontic procedures were not considered due to the posterior location of the tooth and the associated technical challenges. An apical barrier technique using MTA was also considered but not selected because of the difficulty in achieving controlled placement in a tooth with an open apex and limited access. Therefore, an obturation strategy based on a calcium silicate sealer [11] in combination with the piston technique was chosen as a clinically feasible alternative.

Thorough canal disinfection followed by obturation using a calcium silicate–based sealer was associated with radiographic evidence of an adequate apical seal and periapical healing. The favorable clinical and radiographic outcomes may indicate a possible association between the selected obturation strategy and the subsequent resolution of periapical pathology [22].

Limitations of this report include the inherent constraints of a single‐case design, the lack of histological confirmation of tissue response [23], and the absence of long‐term outcome data beyond the 12‐month follow‐up period.

Given the single‐case nature of the present report, further experimental, histological, and comparative studies are needed to determine whether the piston technique provides any measurable advantage over other obturation approaches.

In conclusion, from a clinical perspective, calcium silicate–based sealers used in combination with the piston technique may represent a viable alternative for obturation in selected cases of immature necrotic permanent teeth [24], particularly in posterior regions where conventional apical barrier techniques and regenerative procedures are technically challenging and may be less predictable. Although the present case suggests that the described obturation strategy may be clinically feasible in a selected immature posterior tooth, its interpretation should remain cautious. The technique remains inherently operator‐dependent because the magnitude of apically directed condensation pressure cannot be objectively standardized and depends on the clinician’s tactile feedback and clinical judgment. This may affect reproducibility and increase the risk of sealer extrusion or underfilling. The degree and morphology of the open apex should also be carefully considered. This approach should not be recommended for teeth at earlier stages of root development with markedly divergent apical walls, where the risk of uncontrolled extrusion may be higher. For this reason, minimal pressure should be applied, and intraoperative radiographic assessment should be used to evaluate obturation length, density, and the extent of any sealer extrusion.

## Author Contributions

Conceptualization: Bruno Calkovsky. Methodology: Bruno Calkovsky, Maria Janickova. Investigation: Maria Janickova and Sarah Kalmanova. Data curation: Bruno Calkovsky and Sarah Kalmanova. Writing – original draft: Bruno Calkovsky. Writing – review and editing: Bruno Calkovsky and Rastislav Juricek. Visualization: Sarah Kalmanova and Rastislav Juricek. Supervision: Maria Janickova. Project administration: Bruno Calkovsky.

## Funding

No funding was received for this manuscript.

## Disclosure

All authors have read and approved the final version of the manuscript. Bruno Calkovsky MD had full access to all of the data in this study and takes complete responsibility for the integrity of the data and the accuracy of the data analysis.

## Ethics Statement

The authors have nothing to report.

## Conflicts of Interest

The authors declare no conflicts of interest.

## Data Availability

The data that support the findings of this study are available from the corresponding author upon reasonable request.
